# Assessment of *Helicobacter pylori* positive infected patients according to Clarithromycin resistant 23S rRNA, *rpl22* associated mutations and cyp2c19*1, *2, *3 genes pattern in the Early stage of Gastritis

**DOI:** 10.1186/s13104-022-06227-5

**Published:** 2022-10-25

**Authors:** Atena Abedi Maghami, Ashraf Mohabati Mobarez, Abbas Yadegar, Maryam Nikkhah, Amir Sadeghi, Saber Esmaeili

**Affiliations:** 1 Department of Bacteriology, Faculty of Medical Sciences, Tarbiat Modares University, Al-E Ahmad Exp., Tehran, Iran; 2 Foodborne and Waterborne Diseases Research Center, Research Institute for Gastroenterology and Liver Diseases, Shahid Beheshti University of Medical Sciences, Tehran, Iran; 3 Department of Nanobiotechnology, Faculty of Biological Sciences, Tarbiat Modares University, Tehran, Iran; 4 Gastroenterology and Liver Diseases Research Center, Research Institute for Gastroenterology and Liver Diseases, Shahid Beheshti University of Medical, Sciences, Tehran, Iran; 5 National Reference Laboratory of Plague, Tularemia and Q Fever, Research Centre for Emerging and Reemerging Infectious Diseases, Pasteur Institute of Iran, Tehran, Iran

**Keywords:** Clarithromycin resistant, *Helicobacter pylori*, Homozygote extensive metabolizer, Heterozygote extensive metabolizer, Poor metabolizer

## Abstract

**Objective:**

Clarithromycin resistant *Helicobacter pylori* (CAM-R) is the main cause of standard triple therapy eradicating failure. Proton pump inhibitors (PPIs) directly pose bacteriocidic activity and prepare the optimum condition for Clarithromycin’s best function. In counter with Poor metabolizer subjects, Homozygote Extensive Metabolizers have well characterized by treatment failure. Eventually, determination of CAM-R profile and estimation of PPIs metabolization rate support clinicians in better prescription. So, we explored *Helicobacter pylori*’mutations in 23S rRNA and *rpl22* resistant genes, and *cyp2c19* *1, *2, *3 allele variations, and PPIs metabolization patterns in patients, consequently the results reported to the physician.

**Results:**

Sixteen out of 96 patients considered to be CAM-R *Helicobacter pylori*. A2143C (1/16), *rpl22* insertion (16/16), and GTG deletion (2/16) recorded in CAM-R strains. P450 2C19 human genotyping demonstrated that the highest proportion of the *H. pylori-* positive strains infected patients 43/61(70.49%) categorized in Homozygote extensive metabolizer class. The rest (12/61)19.67% classified as Poor metabolizers, and 6/61(9.83%) distinct from Heterozygote extensive metabolizer group. Proportion of poor metabolizers and Heterozygote extensive metabolizer phenotypes between CAM-R strains mentioned to be 10/16(62.5%), and 6/16(37.5%). Cross points between the most frequently distributed allele in CAM-R strains indicated 81.25% for *2, and ^w^2 for 18.75%.

**Supplementary Information:**

The online version contains supplementary material available at 10.1186/s13104-022-06227-5.

## Introduction

Lower efficient *Helicobacter pylori* eradication by standard triple therapy (STT) has been directly related to the incensement of Clarithromycin resistance rate (CAM-R) [1]. Nevertheless, many compensatory mutations, 23S rRNA related-point mutations A2142G/C, A2143G/C, and recently *rpl22* polymorphisms (GTG deletion and TTCCATGTA insertion) are individually discussed among CAM-R strains [2–4].

Proton pump inhibitors (PPIs) covalently interact with the cysteine residue of proton pumps which inhibit H^+^ releases, thereby collaboration of PPIs in prescription promotes stability and concentration of Clarithromycin. P450 *CYP 2c19* is the liver catabolic enzyme that dominantly corresponds to the metabolization of omeprazole and lansoprazole [5–7]. Among 34 *cyp2c19* allele polymorphisms distinct for the deficiency in drug metabolization, there are three major losses of functions (LOF) cyp2c19*2 (681 G ≥ A), cyp2c19*3(636 G ≥ A), and cyp2c19*17 (806 C ≥ T) in which cyp2c19*2 and cyp2c19*3 are mainly reputed in Asian population than cyp2c19*17 for less than 1% [7].

Based on the *cyp2c19* variants, subjects have been categorized into three groups: Homozygote extensive metabolizer (Hom-EM) with two wild types of allelic polymorphism, Heterozygote extensive metabolizer (Het-EM) with LOF *2 or *3, and poor metabolizer (PM) with two losses of function *2 and *3 [8]. Furuta was the pioneer in the evaluation of *cyp2c19* human genotyping and prediction of cure rate after treatment regime consumption. Beyond the series of randomized clinical trial studies,the eradication rate in the PM group that took the standard dosage of PPIs was considered to be high and in EM participants were reported to be very low, so the presumption of the infection recurrence comes to be high [9, 10].

Thus, to support clinicians in better scheduling, and prevention of drug resistance increasement we performed PCR amplification, and sequencing to evaluate CAM-R related point mutations in 23S rRNA and *rpl22* genes, and Realtime-PCR in the classification of total patients in the early stage of gastritis in *Helicobacter pylori* positive infected individuals, and Clarithromycin resistant strains infected patients based on *cyp2c19* gene mapping.

## Main text

### Material and methods

Total of 96 consenting participants were concluded in this associational study, during the period of April 5th, 2020 to October 9th, 2020. *H. pylori* phenotypically and molecular characterization, bacterial phenotypically antimicrobial drug resistance (ADR), subsequently 23S rRNA and *rpl22* polymorphisms confer CAM-R, determined by PCR amplification, and sequencing that subscribed in Additional files 1 [11, 12] and 2. To detect *cyp2c19* *1, *2 and *3 LOF primers designing were clarified in Additional file 3. Reagents preparation and RT-PCR performance in the differentiation of total patients, *H. pylori-*positive subjects, CAM-R strains infected one according to, *cyp2c19* mentioned variants described in Additional file 4. Additional file 5 (Table S5) have contented *cyp2c19* *1, *2, *3, 23S rRNA, and *rpl22* pair primers list.

## Statistics analysis

The SPSS Statistics for Windows (version 21.0, IBM Corp, Armonk, NY, USA) and Chi-Square (χ2) Definition were applied to search for associations between the variables and classification of the population studied; *p* ≤ 0.05 was considered to be significant range in interpretation.

## Results

Describes the characteristic of the patients from whom *H. pylori* strains were isolated by histopathology test, molecular identification, and bacterial culturing illustrated in Additional file 6. Additional file 7 illustrate the image of early stage of gastritis.

### Cyp2c19*1, *2, and *3 allele distribution

According to our experimental study, frequent allelic polymorphism in the total number of enrolled patients between *cyp2c19* *1(which is the wild type) *2 and *3, denoted for ^w^3 (81.25%), *p* ≤ 0.001. The next were*2 for (13.5%) *p*-value ≤ 0.001, ^w^2(3.125%) *p* ≤ 0.75 and *3(2.08%)* p* ≤ 0.75.

According to the present study, the frequency of *3 and ^w^2 in distribution were the lowest but more than 1%. Chi-Square (χ2) Statistic analysis reports of *cyp2c19* *1 variant distributed in the total patients attended in this work (as the reference group) in comparison to histopathological positive *Helicobacter pylori*, molecular positive *H. pylori*, and culture positive group considered to be significant *p* ≤ 0.001*,* and in CAM-R strains reported for *p* = 0.75. *cyp2c19* *2 allele-span through mentioned classified groups considered to be significant *p* ≤ 0.001 and circulation of *cyp2c19* *3 within total examined subjects noted for *p* ≤ 0.75.

### Accumulation of *cyp2c19* *2 variants among phenotypically and molecular characterized CAM-R strains

**Fig. 1:**
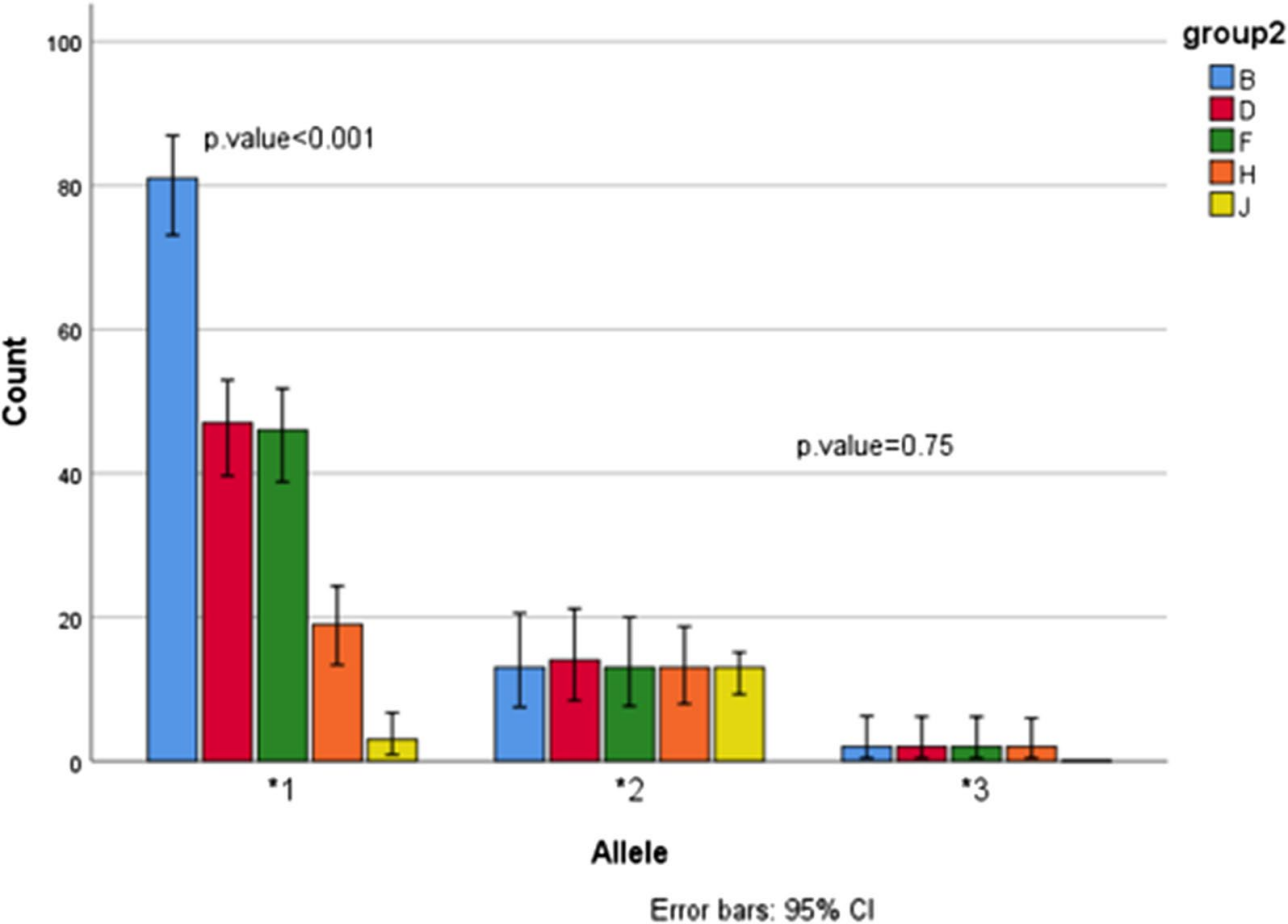
Schematic distribution of *cyp2c19* *1, *2, *3 alleles-frequency. Group (B) recognized as the total gastritis patients, (D)Histopathological examined infected group, (F) Molecular characterized *H. pylori* strains infected patients, (H) culture positive *H. pylori*, and (J) CAM-R strains infected individuals. Statistical report of *cyp2c19* *1 in BDFH considered in significant range *p* ≤ 0.001and in CAM-R strains reported for *p* = 0.75. *cyp2c19* *2 spanning through BDFHJ considered reliable *p* ≤ 0.001. *cyp2c19* *3 circulation in BDFH groups were *p* ≤ 0.75

Distribution of the *cyp2c19* *1, *2, and *3 allelic polymorphism inter culture positive *Helicobacter pylori* strains demonstrated the prevalence of ^w^3(48.57%), *2(37.14%) and ^w^2(8.57%) and *3(5.71%), totally. The dominant allelic-polymorphism through CAM-resistant strains,81.25% was recorded for *2 *p* ≤ 0.001, and 18.75% for ^w^2 *p* ≤ 0.75, respectively. Because of the accumulation of *rpl22* 9 bp insertion, *rpl22* 3 bp deletion, and the only one A2143C point mutation related to CAM-resistance in *2 PM, and *2 Het-EM metabolizer class; therefore, it is clear that the cross point between the most frequent allele that distributed in CAM-R strains will be *2(81.25%), and ^w^2 for 18.75%. The distribution of ^w^3 and *3 among CAM-resistant strains was noted to be zero, Fig. 1.

### Characterization of PPIs catabolization pattern among the patients

The release of our experiment demonstrated that through the total number of patients (n = 96), there were 81.25% distinct for Homozygote extensive PPIs metabolizer with the allelic pattern of (^w^3/^w^3), 6.25% of the patients classified in Het-EM with the allelic pattern of (^w^2/*2) and the rest of 12.49% have been characterized for poor metabolizer category: with the allelic pattern of (*2/*2) for 10.41% and (*3/*3) for 2.08%. The pattern of (^w^3/*3) Het-EM was not obviously detected in our experiment.

### *Cyp2c19* phenotype distribution between infected individuals

Comparative analysis of the total number of the patients demonstrated that 63/96 histopathological examined patients and 61/96 molecular identified patients were *H. pylori* positive. Distribution of the Hom-EM (^w^3/^w^3), Het-EM (^w^2/*2), and PM phenotypes in infected individuals by histopathological and molecular tests reports, were ordinarily 71.42%, 9.52%, 19.04%, and 70.49%,9.83%, and 19.66%. Poor metabolization pattern within the histopathological reported *H. pylori*-positive patients were (*2/*2)15.87%, (*3/*3) 3.17% and for molecular identified patients recorded 16.39% for (*2/*2) and 3.27% for (*3/*3).

### PPIs metabolizer phenotype patterns and profile of CAM-resistant

In the manner of *cyp2c19* gene dosage profiling between CAM-R strains that circulated through the population with the perspective of personalized therapy; replacement of the drug, duration, or drug dosing; first, 35/96 (36.45%) of the individual phenotypically evaluated *Helicobacter pylori* positive, that the prevalence of Hom-EM participants (^w^3/^w^3), Het-EM(^w^2/*2) and PM (*2/*2) vs (*3/*3) were reported 48.57%, 17.14%, 28.57% and 5.71% ordinarily. Details have already accumulated in Table 1. In this survey, there was significant coverage between CAM-resistant strains 16/35(45.71%), and the distribution of two phenotypes of PPIs metabolization rate: 62.5% for PM (*2/*2) and 37.5% for Het-EM (^w^2/*2). The more interesting notification of our results was the accumulation of the total number of the point mutations (A2143C and *rpl22* GTG deletion or 9 bp insertion) correlated with the CAM-R strains in two phenotypes: PM (*2/*2), and Het-EM(^w^2/*2). According to our study from (10/16) 62.5% of poor metabolizer patients were characterized for the allelic pattern of (*2/*2); spanning of CAM-R related point mutations noted to be: 1/10 for A2143C, 8/10 for *rpl22*9bp insertion, and 2/10 for *rpl22* GTG deletion and 9 bp insertion. The molecular pattern of the rest of 6/16 (37.5%) CAM-R isolates with *rpl22* 9 bp insertion, are classified in Het-EM (Table 2).

**Table 1:** Descriptive analysis of p450 2c19 *1, *2, *3 human genomic pattern and allelic variation

*Cyp2c19* polymorphisms	Total number of gastritis patients	Histopathological infected report	*H. pylori* positive molecular report	*H. pylori* culture positive patients	CAM-R *H. pylori*
^w^3/^w^3	78/96(81.25%)	45/63(71.42%)	43/61(70.49%)	17/35(48.57%)	–
*2/*2	10/96(10.41%)	10/63(15.87%)	10/61(16.39%)	10/35(28.57%)	10/16(62.5%)
^w^2/*2	6/96(6.25%)	6/63(9.52%)	6/61(9.83%)	6/35(17.14%)	6/16(37.5%)
*3/*3	2/96(2.08%)	2/63(3.17%)	2/61(3.27%)	2/35(5.71%)	–
*1	84.32%	70.36	75.40%	47.14%	18.75%
*3	2.08%	3.17%	3.27%	5.71%	–
*2	13.5%	20.63%	21.30%	37.14%	81.25%
	N = 96	N = 63	N = 61	N = 35	N = 16

**Table 2:** Cross reaction between *cyp2c19* allelic variations and profile of mutations in CAM R isolates

Target gene	Mutation	PPIs metabolization Phenotype	Metabolization pattern	Total number of CAM-resistant strains
23SrRNA	A2143C transition	PM	*2/*2	1/16
*rpl22*	TTCCATGTA insertion	PM	*2/*2	10/16
*rpl22*	TTCCATGTA insertion	Het-EM	^w^2/*2	6/16
*rpl22*	GTG deletion	PM	*2/*2	2/16

## Discussion

According to Kyoto global consent reports the sensitive, available, rapid, and cost-effective molecular approaches are the health care needed to control the *Helicobacter pylori*’related disease (prophylactic purpose), and improve the cure rate by determination of local CAM-R profile and PPIs-metabolization rate [13–15].

Indeed, this experiment was performed to evaluate the local profile of Clarithromycin resistant strains infected patients consequently cyp2c19*1, *2, *3 patients’ pattern in PPIs (omeprazole and lansoprazole) metabolization rate.

Based on our survey, the most proportion of the patients, histopathological and molecular infected subjects, and culture-positive patients, are classified in the Hom-EM class, that the differences of cyp2c19*1 polymorphisms mention being statistically highly significant (*P* ≤ 0.001). This report is strongly supported by Mahmoudi Saber et al. [16] in Tehran, where the rate of Hom-extensive metabolizer patients was reported for 85.9%. Didevar et al. [17] by investigating the Azari Turkish healthy individuals, demonstrated the most content of the subjects categorized in the Hom-EM group. According to our work, the rest of the patients were classified as PM (12.49%) and Het-EM (6.25%). The report of statistics in cyp2c19*2 mapping, revealed that the differences were more significant than cyp2c19*3, *p* ≤ 0.75. A comprehensive review of the Iranian *cyp2c19* gene Polymorphisms Population reported the spanning of *2 variants (13.6%) that the prevalence was obviously in a row with our work [18], they have been reported *3 allelic variations with the minor allele frequent class (MAF) ≤ 1% that based on our experiment the prevalence of *3 allelic polymorphism described spanning in limited subjects 2.08% *p* ≤ 0.75. The release of our clinical study illustrated that there was no significant relationship between the *cyp2c19* allelic distribution, and the age or gender of the participants, respectively.

Based on our findings, Het-EM patients allelic combination exhibited ^w^2/*2 pattern in diagnosis, which was in order with Saber et al. [19]. In both studies, the association of Het-EM patients with the ^w^3/*3 pattern was considered to be zero. Illustration of the dominant *2/*2 structure of PM patients in our study revealed the similarity to, Saber et al. [16], Namazi et al. [19], Zendehdel et al. [20], and whom reported the relationship between the dominant *2/*2 pattern in PM patients. However, in our experiment, small portion of PM phenotype was considered for *3/*3 pattern, and in their study, such combination was noticed to be zero.

Based on a series of studies exceeded the rate of Clarithromycin resistance up to 15%, and replacement of standard triple therapy by Bismuth quadruple therapy, hybrid (or reverse hybrid) therapy, and concomitant therapy are on the double scale in the prescription [21]. Case-to-case therapy by determination of CAM-R pattern and *cyp2c19* polymorphisms improve the superior in quality, with fewer adverse events [22]. In Turkey, the consequences of CAM-R strains rose rate to 40% linked to decreases in STT efficiency of 55.7% [9]. Choi et al. [22] indicated that 23S rRNA point mutations monitoring in CAM-R strains increase the eradication rate from 82.6% to 91.2%. Nor efficiency improvement but also the rate of eradication-related side effects decreased by 12.0%, which significantly looks different from empirical bismuth quadruple therapy for *H. pylori* first-line eradication regime.

To pretreatment ideally therapy, these findings led us to categorize the patients based on Clarithromycin resistance and pattern of PPIs metabolization rate, and results reported to the physicians. The findings demonstrated that 100% of the phenotypically CAM-R strains are covered by *rpl22* mutations. According to us all the Clarithromycin sensitive patients classified in Homozygote extensive metabolizer group, and all the CAM-resistant patients described for PM 62.5% and Het-EM 37.5%, respectively. It is worth mentioning that accumulation of all CAM-R strains, CAM-R related point mutations in cyp2c19*2 variant were highly significant *p* ≤ 0.001; *rpl22* 9 bp insertion, *rpl22* 3 bp deletion, and the only one A2143C point mutations related to CAM-resistance all categorized in *2 PM, and *2 Het-EM metabolizer class. Yi Song et al. In a similar experience denoted that the distribution of the individuals in Het-EM, EM, and PM groups were 53%, 38%, and 9%; The most interesting notification of their data was the accumulation of the CAM-R related point mutations in EM class [23], that was in counter with our findings.

Based on the results, definition the majority of the individuals and all the CAM-S strains classified in the EM group, by means before the beginning of the therapy, it is so clear that the recurrence of the infection in the subjects that received standard PPIs (dosing or duration) seems to be high. All the CAM-R patients were categorized as PM62.5%, and Het-EM 37.5% groups, which the prediction of cure rate by standard PPIs dosage scheduling in PM groups seems to be fine and controversial in Het-EM group.

### Limitations

For the purpose of tailoring therapy, we introduced three groups of patients to clinicians: first, CAM-R infected subjects (Tagged for 23S rRNA and *rpl22* related point mutations) for drug replacement or alternative treatment regime in use; second, PM patients in CAM-S group (that may suffer from long-term PPIs and side effects), and CAM-R groups; finally, EM patients that the risk of infection recurrence by standard PPIs dose scheduling consider to be very high. Short time for patient follows up project, online system for requesting follow-up appointments, and physician’s persistence in empirical therapy rather than per-patient therapy could be our limitations in this experiment.

## Supplementary Information

**Additional file 1.** The process of *H. pylori* phenotypic and molecular charecterization

**Additional file 2.** Determination of 23S rRNA and *rpl22* polymorphisms confer CAM-R by PCR-amplification and sequencing.

**Additional file 3.** Primer designing.

**Additional file 4.** RT-PCR in *CYP2C19* *1, *2, *3 strain classification.

**Additional file 5: Table S5.** List of cyp2c19 *1, *2, *3 vs 23S rRNA and *rpl22* set primers in polymorphisms designation.

**Additional file 6: Table S5.** The report of Histopathological , molecular, and phenotipic tests in patients evaluation.

**Additional file 7: Figure S7.** Early Stage of Gastritis: Endoscopic Image.

## Data Availability

The data that support the findings of this study are available from the corresponding author upon reasonable request.

## Abbreviations

CAM-R: Clarithromycin resistant
PPIs: Proton pumps inhibitors
STT: Standard triple therapy
LOF: loss of functions
Hom-EM: Homozygote extensive metabolizer
Het-EM: Heterozygote extensive metabolizer
PM: Poor metabolizer
ADR: Antimicrobial drug resistance
WHO: World Health Organization
RCTs: Randomized controlled trials
MAF: Minor allele frequent
