# Supplementary material for: Assessment of Helicobacter pylori positive infected patients according to Clarithromycin resistant 23S rRNA, rpl22 associated mutations and cyp2c19*1, *2, *3 genes pattern in the Early stage of Gastritis
Source: BMC Res Notes. 2022 Oct 25;15:335. doi: 10.1186/s13104-022-06227-5 (PMC9594930; doi:10.1186/s13104-022-06227-5)
Supplement: Supplementary file 1 — Additional file 1. The process of H. pylori phenotypic and molecular charecterization [file 13104_2022_6227_MOESM1_ESM.rtf]

Additional file 1
H. pylori phenotypically and molecular characterization 
Antral biopsies were taken from the patients referred to the Gastroenterology and Hepatology ward of Taleghani hospital, Tehran, Iran. Each specimen was transferred in thioglycolate broth within 5 hours, then immediately grounded, and the homogenized suspension was directly cultured on modified Brucella Agar SÐ Media (Merck, Darmstadt, Germany) with 5% horse blood, 5-10% fetal calf serum (FCS), 2% Skirrow supplement (Quelab, Montreal, Quebec, Canada), and Amphotericin B5 mg/L Sigma-Aldrich (St. Louis, MO, USA). The plates were incubated under microaerophilic conditions for a week. Suspicious colonies were confirmed by bacterial enzymatic activities; Urease, oxidase, and catalase tests. Antimicrobial drug resistance (ADR) was performed by agar dilution method. Briefly, bacterial density was adjusted to be ~ 108 CFU mL−1 and introduced on modified Brucella Agar Sǁ plates (Merck, Darmstadt, Germany) with 10% horse blood, 10% FCS, and Amphotericin B 5 mg/L, and Clarithromycin with the concentration range of 0.016–256 mg/L. Consequently, inoculated plates were incubated under microaerophilic conditions for 3 days. Based on the clinical and laboratory standards institute (CLSI) the cutoff value for the resistance was defined as MICs ≥1 mg/L.DNA extraction was performed in accordance with the Favarogene mini kit (Taiwan) manufacturer's direction, and stored at -20°C until tested.  Subsequently, histopathological reports of the patients (H. pylori positive) were recorded and compared to molecular recognized H. pylori positive infected subjects. 23S rRNA target gene selected to infected subject identification by polymerase chain reaction (PCR). The reagents were prepared in the final volume of 25 ìL made up of 13 ìl of DDW, 8ìl of 2x Master Mix (Amplicon, Spain), 2ìl 23S rRNA F/R primers mixture (Metabion Germany), and finally 2ìl of analyte per tube. Amplification steps follow as initial denaturation at 95°C for 5 minutes, 42 cycles of duplication; denaturation at 95 °C for 60 s, annealing step at 56°C for 45 s, and elongation at 70°C for 60 s. 0.5% agarose gel in a TBE buffer (0.5x), 60 minutes for 90 v was utilized for molecular verification of 147bp amplicon length.
