# Supplementary material for: Assessment of Helicobacter pylori positive infected patients according to Clarithromycin resistant 23S rRNA, rpl22 associated mutations and cyp2c19*1, *2, *3 genes pattern in the Early stage of Gastritis
Source: BMC Res Notes. 2022 Oct 25;15:335. doi: 10.1186/s13104-022-06227-5 (PMC9594930; doi:10.1186/s13104-022-06227-5)
Supplement: Supplementary file 2 — Additional file 2. Determination of 23S rRNA and rpl22 polymorphisms confer CAM-R by PCR-amplification and sequencing. [file 13104_2022_6227_MOESM2_ESM.rtf]

Additional file 2
Determination of 23S rRNA and rpl22 polymorphisms confer CAM-R by PCR-amplification and sequencing 
23S rRNA, rpl22 genes amplification were performed in the total reaction mixture containing 25ìL of the: 12 ìl of DDW, 10ìl master mix (Amplicon, Spain), by addition of 1ìl of 23S rRNA and rpl22 F/R set primers (Metabion Germany) designed in the previous study. 1ìl of the analyte was deposited per tube. The reaction was carried out as follows: initiation denaturation at 95°C for 5 min, 42 cycles of amplification; denaturation at 95°C for 60 s, 23S rRNA annealing temperature at 56°C and rpl22 at 60°C for 35s, consequently extension at 70°C for 60 s. 0.5% agarose gel in a TBE buffer (0.5x), 60 minutes for 90 v was utilized for molecular verification of H pylori 23S rRNA (147bp) and rpl22 (217bp) target-genes. Sanger-sequencing was conducted in a selection of candidate mutations in total H pylori phenotypically characterized CAM-resistant strains.
