# Supplementary material for: Assessment of Helicobacter pylori positive infected patients according to Clarithromycin resistant 23S rRNA, rpl22 associated mutations and cyp2c19*1, *2, *3 genes pattern in the Early stage of Gastritis
Source: BMC Res Notes. 2022 Oct 25;15:335. doi: 10.1186/s13104-022-06227-5 (PMC9594930; doi:10.1186/s13104-022-06227-5)
Supplement: Supplementary file 3 — Additional file 3. Primer designing. [file 13104_2022_6227_MOESM3_ESM.rtf]

Additional file 3
Primer designing
NCBI GenBank database (http://www.ncbi.nlm.nih.gov/genbank) used in pooling sequences of the two most common polymorphic alleles cyp2c19 *2, and*3. cyp2c19*2 (681 G>A, rs 4244285) is located at the fifth exon of human cytochrome P-450 2C19. GenBank record (accession number: AY796203.1) in complete CDS, used as the template in primers designing. cyp2c19*3 (636 G>A, rs 4986893) is located at the fourth exon of human cytochrome P-450 2C19 (CYP2C19). GenBank record: L32982.1 Used as the template in the set -primers designing. Specific R2 primers designated to be fixed in each reaction, so forward primers; F*2, and F2 set to isolate wild-type amplicon 164 bp from mutated one 167bp. In cyp2c19*3 evaluation, F3 primer plotted to be fixed in each reaction, and R*3, R3   pair-primers were included in each reaction in the detection of mutated 149bp from wild-type 152bp. The specificity of pair primers was controlled with the NCBI nucleotide blast database (http://www.ncbi.nlm.nih. gov/blast/). The NCBI primer blast (http://www.ncbi.nlm.nih. gov/primer-BLAST/) was used to organization of the amplification size.
