# Supplementary material for: Assessment of Helicobacter pylori positive infected patients according to Clarithromycin resistant 23S rRNA, rpl22 associated mutations and cyp2c19*1, *2, *3 genes pattern in the Early stage of Gastritis
Source: BMC Res Notes. 2022 Oct 25;15:335. doi: 10.1186/s13104-022-06227-5 (PMC9594930; doi:10.1186/s13104-022-06227-5)
Supplement: Supplementary file 4 — Additional file 4. RT-PCR in CYP2C19 *1, *2, *3 strain classification. [file 13104_2022_6227_MOESM4_ESM.rtf]

Additional file 4
RT-PCR in CYP2C19 *1, *2, *3 strain classification 
To analyze the presumed relationship between cyp2c19 polymorphic variation ~ loss of function (LOF) in Helicobacter pylori positive patients, CAM-R strains infected subjects that phenotypic or genotypic classified according to 23S rRNA or rpl22 associated point mutations; CYP2C19 *1(wild type), *2 and *3 gene- mapping examined among the total of (n=96) human extracted genome from antral biopsies. The RT-PCR assay performed by Rotor-Gene™ 6000 QIAGEN, as follows; initial denaturation at 94˚C for 5 minutes, 38 amplification cycles: 45 sec for the second denaturation at 94˚C, Annealing time for 35 seconds [55˚C for cyp2c19 *2 and 59˚C CYP 2c19 *3], final extension at 70˚C for 45 sec. The Mixture was prepared according to the following direction; 5ìL-Eva green (QIAGEN, USA), 1 ìL of F and R primers (100 ìM -Metabion Germany) were added to 10 ìL PCR grade water (QIAGEN, USA). In the evaluation of cyp2c19 w2 polymorphism; F w2 and in cyp2c19 *2 detection F*2, consequently constant R2 prime added to each reaction. cyp2c19*3 polymorphism was examined for w3 by adding 1 ìL Fw3(constant primer) to 1 ìLRw3, and evaluating mutant 3(*3) by addition of 1 ìL Fw3 / 1 ìL R*3, primer listed in Table 1. Finally, 3ìL of the template was inoculated per reaction. The results were manifested as the curves spotted through the amplification. cyp2c19 *2, *3 primers specificity controlled by the amplification with replacement of extracted DNA S. aureus, E. faecium, C. difficile, and B. fragilis per reaction.
