# Supplementary material for: Assessment of Helicobacter pylori positive infected patients according to Clarithromycin resistant 23S rRNA, rpl22 associated mutations and cyp2c19*1, *2, *3 genes pattern in the Early stage of Gastritis
Source: BMC Res Notes. 2022 Oct 25;15:335. doi: 10.1186/s13104-022-06227-5 (PMC9594930; doi:10.1186/s13104-022-06227-5)
Supplement: Supplementary file 5 — Additional file 5: Table S5. List of cyp2c19 *1, *2, *3 vs 23S rRNA and rpl22 set primers in polymorphisms designation. [file 13104_2022_6227_MOESM5_ESM.rtf]

Additional file 5
Table S5. List of cyp2c19 *1, *2, *3 vs 23S rRNA and rpl22 set primers in polymorphisms designation

a  cyp2c19 *2                                                            Sequence                                                                       Amplicon length      	
RefSeq (AY796203.1)	
  F*2 (MUT)                                      TTCCCACTATCATTGATTATTTCCCA                                 167 bp      	
  F2   (WT)                                         CCACTATCATTGATTATTTCCCGG                                     164 bp	
  R2                                                    TACCTTCTCCATTTTGATCAGGAAGC	
   b cyp2c19 *3	
RefSeq (L32982.1)	
  F3                                                            ACCCTGTGATCCCACTTTCATCC                                                            	
  R*3 (MUT)                                      TTCAGGGGGTGCTTACAATCCTGAT                               149bp              	
  R3   (WT )                                          CCAGGGGGTGCTTACAATCCT                                         152 bp	
      23S rRNA	
  F                                                          ATGAATGGCGTAACGAGATGG	
  R                                                         CGCATGATATTCCCATTAGCAGT                                      147bp	
       rpl22	
  F                                                              TTCTGCGGTCGCTAACGGC	
  R                                                              CTACTTACCTTCTGCTTGATT                                        217bp	

a  Constant reverse primer R2; Flexible Forward set - primers: Forward primer in amplification Cyp2c19 *2 mutation (F*2 MUT), Forward primer in isolation wild type,cyp2c19W2 (F2  WT). 
b  F3: forward constant primer; Flexible reverse set – primers in amplification Cyp2c19*3mutation (R*3 MUT): reverse primer in wild type cyp2c19w3(R3WT) isolation. 
