# Supplementary material for: Assessment of Helicobacter pylori positive infected patients according to Clarithromycin resistant 23S rRNA, rpl22 associated mutations and cyp2c19*1, *2, *3 genes pattern in the Early stage of Gastritis
Source: BMC Res Notes. 2022 Oct 25;15:335. doi: 10.1186/s13104-022-06227-5 (PMC9594930; doi:10.1186/s13104-022-06227-5)
Supplement: Supplementary file 6 — Additional file 6: Table S5. The report of Histopathological , molecular, and phenotipic tests in patients evaluation. [file 13104_2022_6227_MOESM6_ESM.rtf]

Additional file 6
Out of the 96 participants included in this study, (61.45%)59/96 were females, and (38.54%)37/96 were males with an average age of ±42.6 years (16 to 69 years old). Reports of the characteristic of the patients from whom H. pylori strains were isolated by histopathology test, molecular identification, and bacterial culturing displayed to be 63 out of 96 (65%) p-value ≤ 0.001, 61out of 96 (63%) p-value ≤ 0.001, and 35out of 96(36%) p-value ≤ 0.001. Gender differences in histopathological positive H. pylori results, and molecular evaluation, were reported for (52.38%)33/63, (47.61%)30/63, and (54.09% )33/61, (45.90% )28/61 for females and males, ordinarily. According to our data 16/35(45%) were tagged to be CAM-R isolates with the gene bank accession numbers :(MZ677130, MZ677131, MZ677132, MZ677133, MZ677134, MZ677135, MZ677136, MZ677137, MZ677138, MZ825086, MZ825087, MZ677141, MZ677142, MZ677143, MZ677144). In general, classification of CAM-R related point mutations distributed among strains were ordinarily 1/16 (6.2%) for A2143C (MZ677138), 16/16(100%) rpl22 9bp insertion (OK 300075, OK 300076, OK 300077), 2/16 (12.5%) with GTG deletion (OK300079, OK300080), respectively. Among the total participants, no significant differences were demonstrated between the ages or gender of H. pylori positive infected subjects or CAM-R infected groups. 
