# Supplementary figures and images for: Assessment of Helicobacter pylori positive infected patients according to Clarithromycin resistant 23S rRNA, rpl22 associated mutations and cyp2c19*1, *2, *3 genes pattern in the Early stage of Gastritis
Source: BMC Res Notes. 2022 Oct 25;15:335. doi: 10.1186/s13104-022-06227-5 (PMC9594930; doi:10.1186/s13104-022-06227-5)

**Additional file 7**


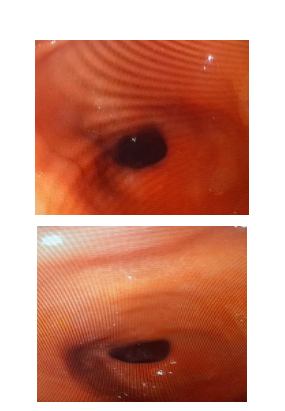


**FigS7.Early Stage of Gastritis: Endoscopic Image**

Supplement: Supplementary file 7 — Additional file 7: Figure S7. Early Stage of Gastritis: Endoscopic Image. [file 13104_2022_6227_MOESM7_ESM.docx]
